# Absolute ranging over 113 km with nanometer precision

**DOI:** 10.1093/nsr/nwaf352

**Published:** 2025-08-22

**Authors:** Yan-Wei Chen, Meng-Zhe Lian, Jin-Jian Han, Ting Zeng, Min Li, Guo-Dong Wei, Yong Wang, Yi Sheng, Ali Esamdin, Lei Hou, Qi Shen, Jian-Yu Guan, Jian-Jun Jia, Ji-Gang Ren, Cheng-Zhi Peng, Qiang Zhang, Hai-Feng Jiang, Jian-Wei Pan

**Affiliations:** Hefei National Research Center for Physical Sciences at the Microscale and School of Physical Sciences, University of Science and Technology of China, Hefei 230026, China; Shanghai Research Center for Quantum Science and CAS Center for Excellence in Quantum Information and Quantum Physics, University of Science and Technology of China, Shanghai 201315, China; Hefei National Laboratory, University of Science and Technology of China, Hefei 230088, China; Hefei National Research Center for Physical Sciences at the Microscale and School of Physical Sciences, University of Science and Technology of China, Hefei 230026, China; Shanghai Research Center for Quantum Science and CAS Center for Excellence in Quantum Information and Quantum Physics, University of Science and Technology of China, Shanghai 201315, China; Hefei National Laboratory, University of Science and Technology of China, Hefei 230088, China; Hefei National Research Center for Physical Sciences at the Microscale and School of Physical Sciences, University of Science and Technology of China, Hefei 230026, China; Shanghai Research Center for Quantum Science and CAS Center for Excellence in Quantum Information and Quantum Physics, University of Science and Technology of China, Shanghai 201315, China; Hefei National Laboratory, University of Science and Technology of China, Hefei 230088, China; Hefei National Research Center for Physical Sciences at the Microscale and School of Physical Sciences, University of Science and Technology of China, Hefei 230026, China; Shanghai Research Center for Quantum Science and CAS Center for Excellence in Quantum Information and Quantum Physics, University of Science and Technology of China, Shanghai 201315, China; Hefei National Laboratory, University of Science and Technology of China, Hefei 230088, China; Hefei National Research Center for Physical Sciences at the Microscale and School of Physical Sciences, University of Science and Technology of China, Hefei 230026, China; Shanghai Research Center for Quantum Science and CAS Center for Excellence in Quantum Information and Quantum Physics, University of Science and Technology of China, Shanghai 201315, China; Hefei National Laboratory, University of Science and Technology of China, Hefei 230088, China; Hefei National Research Center for Physical Sciences at the Microscale and School of Physical Sciences, University of Science and Technology of China, Hefei 230026, China; Shanghai Research Center for Quantum Science and CAS Center for Excellence in Quantum Information and Quantum Physics, University of Science and Technology of China, Shanghai 201315, China; Hefei National Laboratory, University of Science and Technology of China, Hefei 230088, China; Xinjiang Astronomical Observatory, Chinese Academy of Sciences, Urumqi 830011, China; Hefei National Research Center for Physical Sciences at the Microscale and School of Physical Sciences, University of Science and Technology of China, Hefei 230026, China; Shanghai Research Center for Quantum Science and CAS Center for Excellence in Quantum Information and Quantum Physics, University of Science and Technology of China, Shanghai 201315, China; Hefei National Laboratory, University of Science and Technology of China, Hefei 230088, China; Xinjiang Astronomical Observatory, Chinese Academy of Sciences, Urumqi 830011, China; Hefei National Research Center for Physical Sciences at the Microscale and School of Physical Sciences, University of Science and Technology of China, Hefei 230026, China; Shanghai Research Center for Quantum Science and CAS Center for Excellence in Quantum Information and Quantum Physics, University of Science and Technology of China, Shanghai 201315, China; Hefei National Laboratory, University of Science and Technology of China, Hefei 230088, China; Hefei National Research Center for Physical Sciences at the Microscale and School of Physical Sciences, University of Science and Technology of China, Hefei 230026, China; Shanghai Research Center for Quantum Science and CAS Center for Excellence in Quantum Information and Quantum Physics, University of Science and Technology of China, Shanghai 201315, China; Hefei National Laboratory, University of Science and Technology of China, Hefei 230088, China; Hefei National Research Center for Physical Sciences at the Microscale and School of Physical Sciences, University of Science and Technology of China, Hefei 230026, China; Shanghai Research Center for Quantum Science and CAS Center for Excellence in Quantum Information and Quantum Physics, University of Science and Technology of China, Shanghai 201315, China; Hefei National Laboratory, University of Science and Technology of China, Hefei 230088, China; Hefei National Laboratory, University of Science and Technology of China, Hefei 230088, China; Key Laboratory of Space Active Opto-Electronic Technology, Shanghai Institute of Technical Physics, Chinese Academy of Sciences, Shanghai 200083, China; Hefei National Research Center for Physical Sciences at the Microscale and School of Physical Sciences, University of Science and Technology of China, Hefei 230026, China; Shanghai Research Center for Quantum Science and CAS Center for Excellence in Quantum Information and Quantum Physics, University of Science and Technology of China, Shanghai 201315, China; Hefei National Laboratory, University of Science and Technology of China, Hefei 230088, China; Hefei National Research Center for Physical Sciences at the Microscale and School of Physical Sciences, University of Science and Technology of China, Hefei 230026, China; Shanghai Research Center for Quantum Science and CAS Center for Excellence in Quantum Information and Quantum Physics, University of Science and Technology of China, Shanghai 201315, China; Hefei National Laboratory, University of Science and Technology of China, Hefei 230088, China; Hefei National Research Center for Physical Sciences at the Microscale and School of Physical Sciences, University of Science and Technology of China, Hefei 230026, China; Shanghai Research Center for Quantum Science and CAS Center for Excellence in Quantum Information and Quantum Physics, University of Science and Technology of China, Shanghai 201315, China; Hefei National Laboratory, University of Science and Technology of China, Hefei 230088, China; Hefei National Research Center for Physical Sciences at the Microscale and School of Physical Sciences, University of Science and Technology of China, Hefei 230026, China; Shanghai Research Center for Quantum Science and CAS Center for Excellence in Quantum Information and Quantum Physics, University of Science and Technology of China, Shanghai 201315, China; Hefei National Laboratory, University of Science and Technology of China, Hefei 230088, China; Hefei National Research Center for Physical Sciences at the Microscale and School of Physical Sciences, University of Science and Technology of China, Hefei 230026, China; Shanghai Research Center for Quantum Science and CAS Center for Excellence in Quantum Information and Quantum Physics, University of Science and Technology of China, Shanghai 201315, China; Hefei National Laboratory, University of Science and Technology of China, Hefei 230088, China

**Keywords:** precision metrology, optical frequency comb, high-precision long-distance absolute ranging

## Abstract

Accurate long-distance ranging is crucial for diverse applications, including satellite formation flying, very-long-baseline interferometry, gravitational-wave observations, geographical research, etc. The integration of the time-of-flight measurement with phase interference in the dual-comb method enables high-precision ranging with a rapid update rate and an extended ambiguity range. Pioneering experiments have achieved remarkable absolute ranging precision over short paths. However, achieving similar precision over longer distances remains technically challenging due to high transmission loss and noise. In this article, we propose a bistatic dual-comb ranging approach that enables successful ranging over a distance of 113 km. We employ air dispersion analysis and a synthetic repetition rate technique to extend the ambiguity range of the inherently noisy channel beyond 100 km. The achieved ranging precision is 11.5 $\mu$m @ 1.3 ms, 681 nm @ 1 s and 82 nm @ 21 s, as confirmed through a comparative analysis of two independent systems. The advanced long-distance ranging technology is expected to have immediate implications for space research initiatives, such as the space telescope array and satellite gravimetry.

## INTRODUCTION

Achieving precise long-distance ranging requires a delicate balance between an extended ambiguity range and high precision. Precise ranging relies on accurate timing to determine distance. Interferometry methods, such as continuous-wave laser interferometry, achieve sub-nanometer resolution by measuring the phase change of an optical signal along its path [1–3]. However, due to the periodicity of the phase, the ambiguity range is limited to half a wavelength. Laser ranging techniques based on pulsed or radio-frequency-modulated signals, as well as the microwave ranging based on Global Navigation Satellite System (GNSS) signals, can achieve a larger ambiguity range through time-of-flight measurements [4–11]. Nevertheless, because of the constraints imposed by phase and time resolution, their best precision is only of the order of sub-millimeters.

In principle, the synchronization of phases from radio frequencies to optical frequencies enables the achievement of both high precision and a wide ambiguity range. This is crucial for certain applications, such as satellite formation flying, black hole imaging, tests of general relativity and geographical research [12–23]. For example, to achieve a large synthetic aperture in the X-ray band, the measurement of the absolute baseline distance must be obtained with nanoscale precision [12–14].

Optical frequency combs (OFCs) effectively fulfill this requirement by serving as efficient gears that establish connections between radio frequencies and optical frequencies [24]. In recent advancements, researchers have explored various ranging methods based on OFCs [25–37]; however, only a few of them have been demonstrated over long-distance outdoor paths [31–33]. The dual-comb ranging method, which leverages the phase interference between adjacent teeth of OFCs, exhibits the potential to extend the ambiguity range beyond 100 km. The phase locking of OFCs with a stable source, such as an ultra-stable laser, is anticipated to achieve accuracy in the tens of nanometers simultaneously, with a fractional ranging resolution of about ${10}^{-13}$. However, due to high transmission loss and noise (atmospheric disturbance), it is extremely challenging to measure such a long distance with the accuracy expected by theory. So far, the reported longest absolute distance measurement based on dual-comb ranging is still less than 1 km [32].

## PRINCIPLE

**Figure 1.**
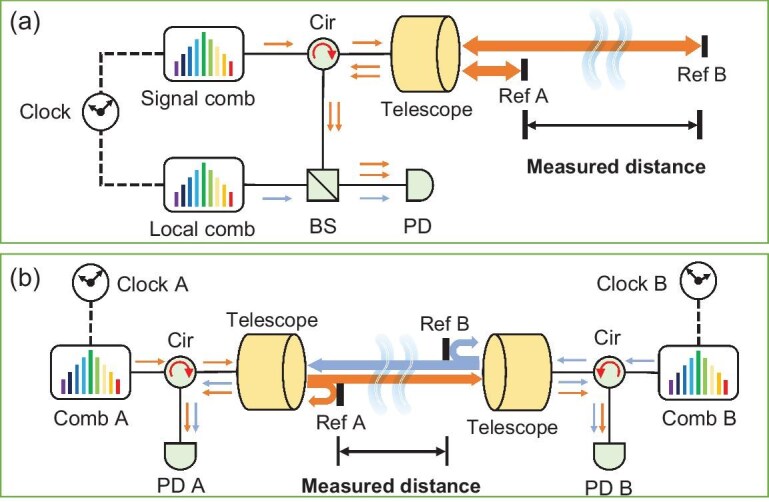
Two methods of dual-comb absolute ranging. (a) Monostatic method: the light from the signal comb is reflected by both reference plane A and reference plane B, resulting in interference with the local comb to obtain distance information. (b) Bistatic method: comb A and comb B are individually phase locked to the local clock. The interference between the light reflected from the local reference plane and the light transmitted from the opposite end is utilized for extracting distance information. In each absolute distance measurement, clock A and clock B are synchronized through time-frequency transfer [38] using the same setup.

In this work, we propose a bistatic dual-comb ranging (BDCR) approach to extend the ranging distance by mitigating the effects of transmission noise and loss. The traditional monostatic dual-comb ranging method and BDCR are shown in Fig. 1(a) and 1(b), respectively. In the monostatic method, the light from the signal comb is reflected by both reference plane A and reference plane B, resulting in interference with the local comb to obtain distance information. In the bistatic method, comb A and comb B are individually phase locked to the local clock. The interference between the light reflected from the local reference plane and the light transmitted from the opposite end is utilized for extracting distance information.

**Figure 2.**
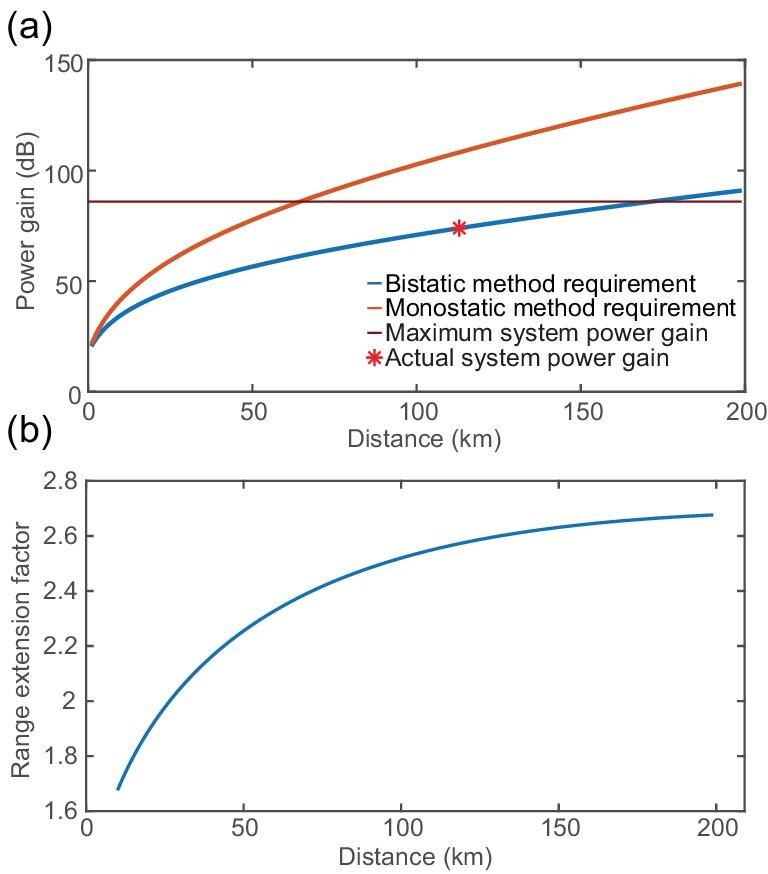
The advantage of BDCR. (a) The required power gain for the bistatic method and the monostatic method at various distances, as well as the maximum and actual power gains of our system. (b) The relationship between the range extension factor and the distance (measured by the bistatic method). Here, the range extension factor is defined as the ratio between the measured distance obtained using the bistatic method and that obtained using the monostatic method.

Compared to the monostatic method, the advantage of the bistatic method lies in the fact that the traveling signal only passes the distance once and is not limited by the diameter of the remote reflector (Ref B in Fig. 1(a)), thus avoiding the power loss of the signal outside the reflector [39]. The power gains required at different distances for both methods are depicted in Fig. 2(a). In our experiment, the actual required power gain for a 113 km path is 74 dB [38]. As shown in Fig. 2(b), for distances exceeding 100 km, the bistatic approach can extend the range by at least 2.5 times with identical signals and detection sensitivity. This bistatic method increases complexity of the setup and data extraction process due to the additional timing combinations on both ends and the need for prior time alignment using time-frequency transfer [38]. However, this choice can be acceptable for certain applications primarily influenced by power loss. For instance, in satellite gravimetry, the detection of Earth’s gravitational field can be achieved through inter-satellite ranging between Geostationary Earth Orbit (GEO) and Low Earth Orbit (LEO) satellites [22]. Based on the parameters of our current experimental setup, the power loss of the monostatic method is 106 dB, while the power loss of the bistatic method is 53 dB. Therefore, in this application, the power loss requirement of the monostatic method is difficult to meet, and the bistatic method is suitable.

In the BDCR, terminals A and B are equipped with OFCs that synchronize to stable clocks. These two OFCs have slightly different repetition rates (a few kilohertz) to ensure regular interference. At each terminal, a telescope is employed for receiving and transmitting OFC signals. A fraction of the launched OFC signal is reflected by a reference mirror, serving as the local reference, while the other part traverses the distance to be measured and is collected by the telescope at the other terminal. Interference occurs between the local reference and the traveling signal at each terminal. The distance to be measured refers to the length between two reference mirrors. The timing results extracted from the interferograms at terminals A and B are expressed as ${T}_{A} = T_{L} + {\tau }_{BA}$ and ${T}_{B} = T_{L} - {\tau }_{BA}$, where $T_{L}$ represents the flight time of light between two reference mirrors, and ${\tau }_{BA}$ denotes the time difference between clocks at terminals A and B. The distance can be determined from the timing data, the velocity of light *c* and the refractive index of the medium *n*. Subsequently, the distance is calculated as $L = c({T}_{A} + {T}_{B})/{2n}$.

## SETUP

**Figure 3.**
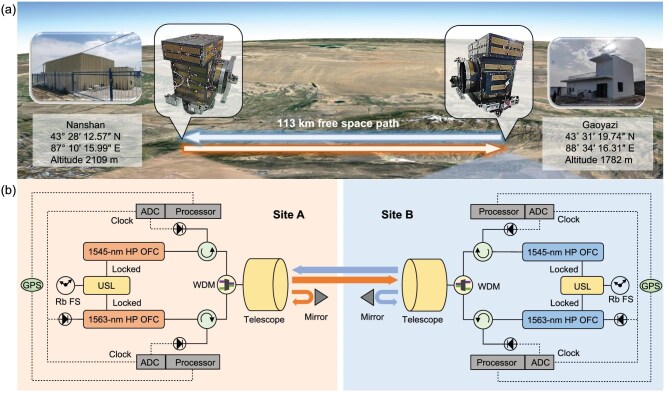
The experimental setup. (a) Overview of the 113 km experimental path for BDCR. (b) The primary apparatus at two experimental sites. The OFCs at the two terminals of the same wavelength have a slight frequency difference, forming a set of equipment for BDCR. The performance of the BDCR method is evaluated by comparing the results of two independent ranging devices at different wavelengths. USL, ultra-stable laser; HP OFC, high-power optical frequency comb; WDM, wavelength-division multiplexer; GPS, global positioning system; ADC, analog-to-digital converter; Rb FS, rubidium frequency standard.

We conducted a ranging experiment at a distance of 113 km to validate the BDCR approach in Urumqi, Xinjiang Province. The performance of the BDCR method was evaluated by comparing the results of two independent ranging devices at different wavelengths. Figure 3(a) illustrates the location information, while Fig. 3(b) depicts the experimental setup. Both terminals are equipped with identical instruments, including an ultra-stable laser with a stability of 3E-15 @ 1 s, two high-power (1 W) OFCs with wavelengths centered at 1545 nm and 1563 nm, respectively, and a telescope with an aperture diameter of 400 mm. In each terminal, both OFCs with different wavelengths are phase locked to the ultra-stable laser. In our experimental design, the repetition frequencies of the OFCs are uniquely determined by the frequency of the ultra-stable laser at each terminal. The frequency difference of the ultra-stable lasers located at both terminals can be initially controlled within tens of hertz using time-frequency dissemination [38]. The relative drift of the frequency difference is about 0.1 Hz/s, which has a negligible influence on ranging accuracy during the data acquisition time.

The OFC signals of different wavelengths are combined and separated at each terminal by using a wavelength-division multiplexer (WDM). A common-mode path is established between the WDMs for the two BDCR systems operating at different wavelengths. In this path, a corner reflector with a diameter of 2 in. is positioned at a distance of 0.1 m from the telescope to serve as the reference for ranging at each terminal. The OFC signal reflected by the corner reflector and that transmitted from the opposite terminal are wavelength-dependently guided by the WDMs at each terminal and finally coupled into the corresponding pigtail collimators.

In addition to using the BDCR method, high-power OFCs and large-aperture telescopes, we also utilize low-noise photodetectors (PDs) and accurate data acquisition and processing systems to overcome the challenges of high power loss. The low-noise PDs receive approximately 20 $\mu$W of OFC signal reflected from the local corner reflector and approximately 40 nW of OFC signal from the opposite terminal. The received power of 40 nW aligns well with the estimated path loss. The equivalent noise power of the PD is 3.5 pW$/\sqrt{\rm Hz}$, enabling a high detection sensitivity below 3 nW. High-performance data acquisition and processing systems enable us to obtain high-precision ranging results from phase interference at the low signal-to-noise ratio.

Another challenge lies in achieving a substantial ambiguity range in the presence of high noise caused by air turbulence and atmospheric changes along the 113 km free-space path. The time deviation of the time of flight over the open-air path was measured at approximately 69 fs @ 1 s and 3.5 ps @ 300 s, while the time jitter ranges between 40–60 fs [38]. By comparing the results of two independent OFC interference systems, we can effectively capture real-time atmospheric drift and mitigate its impact. The ambiguity range depends on the period of the OFC signal through the path [28]. In our experiments, the OFC signals at both sites passing through the path had a small difference in repetition frequency, resulting in two ambiguity ranges, denoted ${D}_{r1}$ and ${D}_{r2}$. They can be determined as ${D}_{r1}=c/2n{f}_{r}$ and ${D}_{r2}=c/2n({f}_{r}+\Delta {f}_{r})$. To extend the ambiguity range, we employed the ‘synthetic repetition rate’ technique and conduct an air dispersion analysis.

The ‘synthetic repetition rate’ technique is similar to that of the ‘synthetic wavelength’ technique [28,32]. By utilizing two combinations of repetition frequencies, ${f}_{r}$ and ${f}_{r}+\Delta {f}_{r}$, as well as ${f}_{r}$ and ${f}_{r}-\Delta {f}_{r}$, for OFCs with identical wavelengths at both terminals, and by exchanging the two repetition frequencies in each combination between the two terminals, we are able to achieve an extended ambiguity range, denoted ${D}_{AR}=({D}_{r1}{D}_{r2}) / [2({D}_{r2}-{D}_{r1})]$. The ranging distance *L* within the interval (0, ${D}_{AR}$) can be determined using the formula ${L}={N}_{1}{D}_{r1}/2+{d}_{1}={N}_{2}{D}_{r2}/2+{d}_{2}$, where $N_{1}$ and $N_{2}$ represent the period numbers, and ${d}_{1}$ and $d_{2}$ denote distances obtained from the interference waveforms within the ranges (0, ${D}_{r1}/2$) and (0, ${D}_{r2}/2$). On the other hand, the exchange in each combination effectively cancels additional ranging asymmetry caused by non-common fiber paths at a sub-meter level. Based on the broad wavelength feature of OFCs and the air refractive index model [40], we use the air dispersion analysis to achieve a preliminary ranging result from the phase information of the interferogram, with an ambiguity range of about ${10}^{8}$ km and a precision within 2 km (see the online supplementary material for details). The air dispersion analysis provides a rough ranging value of $113 \pm 2$ km.

## RESULTS AND DISCUSSION

**Figure 4.**
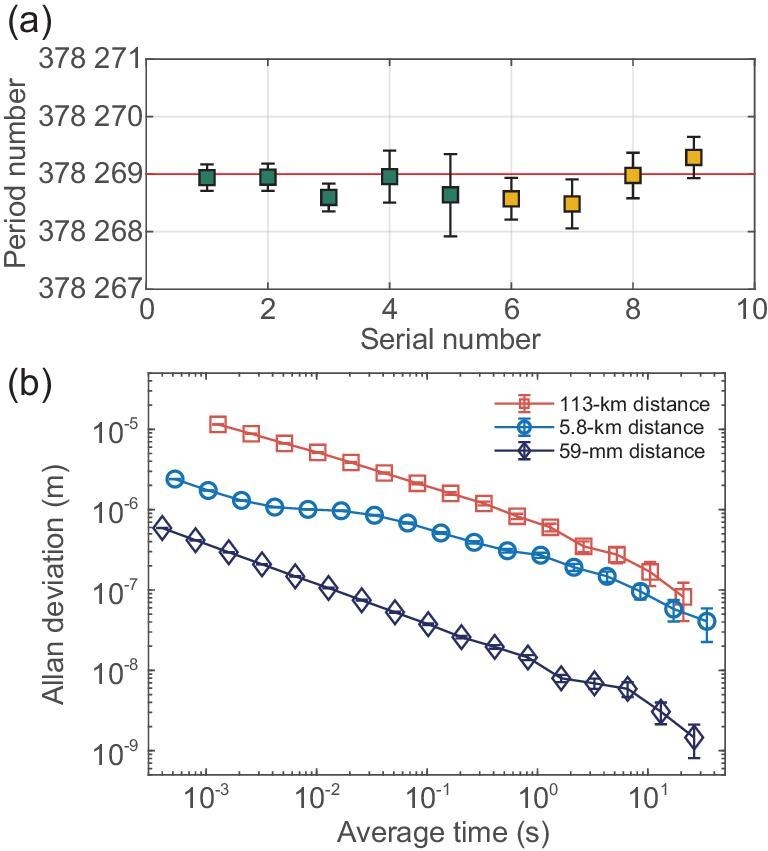
Experimental results of BDCR. (a) Period number $N_{1}$ of absolute ranging. The absolute distance *L* is defined as ${L}={N}_{1}{D}_{r1}/2+{d}_{1}={N}_{2}{D}_{r2}/2+{d}_{2}$, where ${D}_{r1}$ and ${D}_{r2}$ are determined by the parameters of the OFCs, and ${d}_{1}$ and ${d}_{2}$ are obtained from the interference waveforms within the ranges (0, ${D}_{r1}/2$) and (0, ${D}_{r2}/2$), respectively. Here ${N}_{2}$ is equal to ${N}_{1}+4$, as defined by the preliminary ranging result. Each period (${D}_{r1}/2$) or (${D}_{r2}/2$) corresponds to an approximate distance of 0.3 m. The data were collected over two consecutive days between 8 p.m. and 10 p.m., with each day represented by the same color. (b) Precision of absolute ranging. The red open square, blue open circle and purple diamond lines represent the results over paths of 113 km, 5.8 km and 59 mm, respectively. Here, the ranging precision of 59 mm achieved from indoor measurements represents the system’s floor. The ranging precision results of 5.8 and 113 km were obtained in open-air environments.

The key to obtaining an absolute distance lies in acquiring integer period values $N_{1}$ and $N_{2}$ through the use of the synthetic repetition rate technique. In our specific case, the extended ambiguity range is limited to about 60 km due to the influence of residual atmospheric noise on the measurement accuracy. Here, we have set ${D}_{AR}$ as 30 km, which is shorter than 60 km but longer than the resolution required for air dispersion analysis. The coarse distance value of 113 km obtained from the air dispersion analysis indicates that $N_{2}$ is equal to $N_{1}+4$. By utilizing the equation ${L}={N}_{1}{D}_{r1}/2+{d}_{1}={N}_{2}{D}_{r2}/2+{d}_{2}$ and employing two sets of experimentally obtained $\lbrace d_{1}\rbrace$ and $\lbrace d_{2}\rbrace$, the period number $N_{1}=(2D_{r2}+d_{2}-d_{1})/(D_{r1}/2-D_{r2}/2)$ is accurately determined, as illustrated in Fig. 4(a). The average value observed is 378 268.82 with a standard deviation of 0.26. It should be noted that all period values $N_{1}$ are consistently around 378 269, as the data were collected over consecutive days between 8 p.m. and 10 p.m.

The ranging precision achieved by comparing the results of two independent OFC interferences is shown in Fig. 4(b). The Allan deviation for 113-km ranging is 11.5 $\mu$m @ 1.3 ms, 681 nm @ 1 s, and 82 nm @ 21 s. In order to test the system, we also conducted ranging experiments over distances of 59 mm and 5.8 km using the same equipment. The precision for the outdoor path of 5.8 km achieves 274 nm @ 1 s and 41 nm @ 34 s. The system’s floor, obtained in the 59-mm ranging experiments, is 12 nm @ 1 s and 1.5 nm @ 26 s. Precision decreases with increasing distance due to the gradual accumulation of atmospheric noise along the measurement path. Additionally, data rates drop with distance, leading to larger errors.

**Figure 5.**
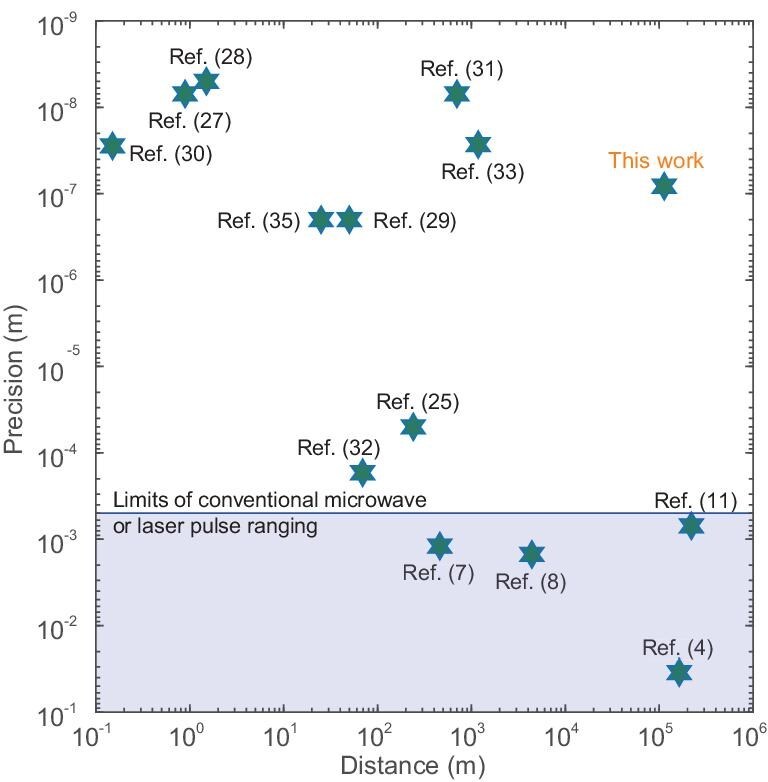
High-precision absolute ranging results by different methods. The results in the gray area are based on techniques for modulating microwave and laser pulse ranging, while other results are obtained using various OFC methods.

The experimental results of various ranging methods are presented in Fig. 5. The BDCR approach demonstrates the capability to achieve absolute ranging on a 100-km scale with sub-micrometer precision. Compared with other methods, BDCR significantly improves the absolute distance measurement accuracy by more than several orders of magnitude at the equivalent measurement length, which is crucial for space research initiatives. For instance, the accurate positioning of formation flying satellites in the micro-arcsecond X-ray imaging mission requires absolute ranging with sub-micrometer accuracy [12–14]. The length of the inter-satellite baseline directly impacts the angular resolution of the telescope. Our approach is expected to increase the length of the baseline beyond 100 km, resulting in an angular resolution of about ${10}^{-9}$ arcsec, which is three orders of magnitude better than the finest resolution images ever achieved (in the radio part of the spectrum). Furthermore, accurate inter-satellite absolute ranging enables precise satellite positioning and orbit tracking, which is a significant addition to satellite gravimetry [21–23]. Compared to existing interferometry methods, our approach offers the advantage of avoiding cycle slips in inter-satellite ranging results. This allows us to instantaneously capture the complete variations of the Earth’s gravity field during large-scale changes in geology, such as earthquakes, floods and volcanic eruptions.

**Figure 6.**
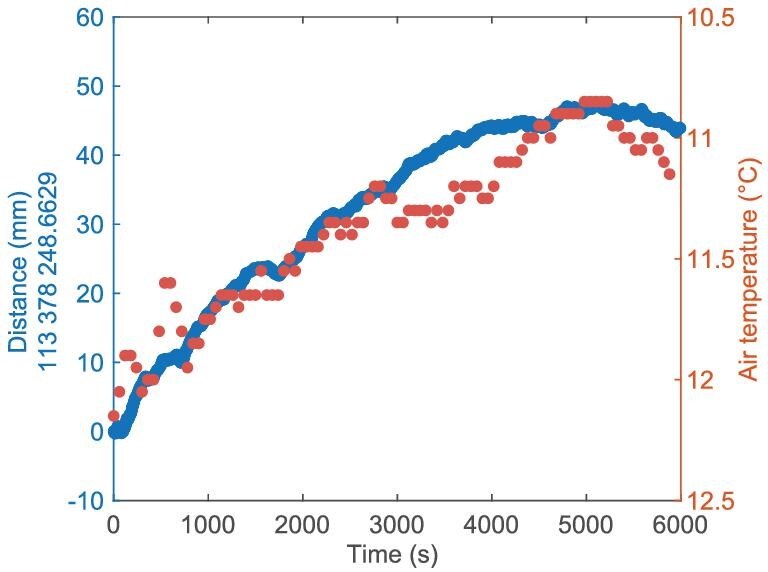
Long-term ranging results of BDCR. By combining the measured values ${d}_{1}$ at each moment, the calculated values ${N}_{1}$ and the average meteorological data, the absolute distance values *L* can be obtained. The distance results (blue) show an initial value of 113 378 248.6629 mm with an overall fluctuation of 50 mm. The average temperatures (red) obtained from the two weather stations also exhibit a similar trend to the observed variation in distance.

By combining the measured values ${d}_{1}$ at each moment, the calculated values ${N}_{1}$ and the meteorological data [40], the absolute distance values *L* can be obtained. Figure 6 shows the 6000-s absolute ranging results. We monitor meteorological conditions with two weather stations (PC-8) located at each terminal. As previously mentioned, ranging is based on measuring the time of flight, so its accuracy is limited by uncertainties in measuring the air refractive index at a level of approximately ${10}^{-7}$. Further improvements can be achieved through the use of dual-comb spectroscopy [39], which accurately determines certain atmospheric conditions, or the two-color method [41], which does not rely on environmental parameters. Fortunately, the space telescope array and satellite gravimetry operate in space at least 400 km above Earth’s surface, experiencing an extremely low pressure level of ${10}^{-8}$ Pa. The uncertainty associated with the air refractive index in these space applications is below ${10}^{-16}$, thereby enabling our systems to attain a fractional ranging uncertainty of ${7.3}\times {10}^{-13}$.

## CONCLUSION

In summary, to achieve high-precision absolute ranging at the 100-km level, we propose a novel BDCR method that extends the ranging distance by mitigating the effects of transmission noise and loss. Compared to the traditional monostatic dual-comb ranging method, this innovative approach significantly reduces power loss, enabling a 2.5-fold extension in measuring distance, thus supporting the exploration of the limits of absolute ranging. In addition, to demonstrate a high-precision absolute distance measurement experiment over 100 km, we utilized high-power OFCs, large-aperture telescopes, low-noise photodetectors, and precise data acquisition and processing systems in our experiments. We employ a combination of air dispersion analysis and the synthetic repetition rate method to overcome transmission noise. The air dispersion analysis offers an ambiguity range of ${10}^{8}$ km with a resolution of 2 km, while the synthetic repetition rate method provides nanometer precision along with an ambiguity range of 30 km. By using these techniques, we have demonstrated high-precision absolute distance measurements over a path of 113 km with a precision of 82 nm @ 21 s. To the best of our knowledge, this study represents the first instance of such precise absolute distance measurement over a path exceeding 100 km. However, the assessment of system accuracy is based on the assumption that the refractive index data are accurate and the uncertainty in the refractive index remains the most significant limiting factor for ground-based ranging. To effectively overcome this limitation, BDCR is planned for use in atmospheric-free environments, like future inter-satellite absolute distance measurements. This technology is expected to improve the angular resolution of space telescope arrays and prevent the cycle slips in satellite gravimetry.

## Supplementary Material

nwaf352_Supplemental_File
