# Supplementary material for: Absolute ranging over 113 km with nanometer precision
Source: Natl Sci Rev. 2025 Aug 22;12(11):nwaf352. doi: 10.1093/nsr/nwaf352 (PMC12629240; doi:10.1093/nsr/nwaf352)
Supplement: nwaf352_Supplemental_File [file nwaf352_supplemental_file.pdf]

Supplementary Information for
113 km absolute ranging with nanometer precision

Yan-Wei Chen[1,2,3,†], Meng-Zhe Lian[1,2,3,†], Jin-Jian Han[1,2,3], Ting Zeng[1,2,3], Min Li[1,2,3], Guo-Dong Wei[1,2,3], Yong Wang[4], Yi Sheng[1,2,3], Ali Esamdin[4], Lei Hou[1,2,3], Qi Shen[1,2,3], Jian-Yu Guan[1,2,3], Jian-Jun Jia[3,5], Ji-Gang Ren[1,2,3], Cheng-Zhi Peng[1,2,3], Qiang Zhang[1,2,3,*], Hai-Feng Jiang[1,2,3,*], and Jian-Wei Pan[1,2,3,*]

[1]Hefei National Research Center for Physical Sciences at the Microscale and School of Physical Sciences, University of Science and Technology of China, Hefei 230026, China

[2]Shanghai Research Center for Quantum Science and CAS Center for Excellence in Quantum Information and Quantum Physics, University of Science and Technology of China, Shanghai 201315, China

[3]Hefei National Laboratory, University of Science and Technology of China, Hefei 230088, China

[4]Xinjiang Astronomical Observatory, Chinese Academy of Sciences, Urumqi 830011, China

[5]Key Laboratory of Space Active Opto-Electronic Technology, Shanghai Institute of Technical Physics, Chinese Academy of Sciences, Shanghai 200083, China

[†]These authors contributed equally to this work. [*] Corresponding authors.

1 Principle of the BDCR

In the BDCR, the accurate time and distance information is achieved by using linear optical sampling (LOS) [3]. In the LOS, the electric fields of OFCs in terminals A and B are expressed as:

$$\begin{aligned}
E_{AP}(t) &= \sum_k E_{A,k} \exp\left[i2\pi\left(\nu_A + kf_A\right)\left(t - T_{CRA} - T_L - T_{DRB}\right)\right] \\
E_{AR}(t) &= \sum_k E_{A,k} \exp\left[i2\pi\left(\nu_A + kf_A\right)\left(t - T_{CRA} - T_{DRA}\right)\right] \\
E_{BP}(t) &= \sum_k E_{B,k} \exp\left[i2\pi\left(\nu_B + kf_B\right)\left(t - T_{CRB} - T_L - T_{DRA} - \tau_{BA}\right)\right] \\
E_{BR}(t) &= \sum_k E_{B,k} \exp\left[i2\pi\left(\nu_B + kf_B\right)\left(t - T_{CRB} - T_{DRB} - \tau_{BA}\right)\right]
\end{aligned} \tag{1}$$

where the electric field $E_{AP}(t)$ represents the portion of the comb A laser that arrives at terminal B, while $E_{AR}(t)$ represents the portion of the comb A laser that is reflected from the local reference in terminal A. Similarly, $E_{BP}(t)$ and $E_{BR}(t)$ represent corresponding components of the comb B laser. The variables T_{CRA}, T_{DRA}, T_{CRB} and T_{DRB} denote the flight time from the local OFC to the local reference plane and from the local reference plane to the detector at terminal A and terminal B, respectively. The clock difference between terminals A and B is τ_{BA}. The flight time of the distance between the two reference planes is defined as T_L. The repetition frequencies f_A and f_B are approximately equal to f_r with a slight difference Δf_r. ν_A and ν_B denote the frequencies of the comb teeth closest to the ultra-stable laser at each site. Interference signals are detected using photodetectors (PDs). The voltage output of the PD in terminal A is:

$$\begin{aligned}
&V_A(t) \propto \mathrm{Im}\left[E^*_{AR}(t)E_{BP}(t)\right] = \mathrm{Im}\{\exp\left[i2\pi\left(\nu_B - \nu_A\right)t\right] \\
&\times \exp\left[i2\pi\nu_A\left(T_{CRA} + T_{DRA}\right) - i2\pi\nu_B\left(T_{CRB} + T_L + T_{DRA} + \tau_{BA}\right)\right] \\
&\times \sum_k E_{A,k}E_{B,k} \exp\left[i2\pi k\Delta f_r t + i2\pi kf_A\left(T_{CRA} + T_{DRA}\right) - i2\pi kf_B\left(T_{CRB} + T_L + T_{DRA} + \tau_{BA}\right)\right]\}
\end{aligned} \tag{2}$$

while the voltage output of the PD in terminal B is:

$$\begin{aligned} V_B(t) &\propto \mathrm{Im}\left[E_{AP}^*(t) E_{BR}(t)\right] = \mathrm{Im}\{\exp\left[i2\pi\left(\nu_B - \nu_A\right)t\right] \\ &\times \exp\left[i2\pi\nu_A\left(T_{CRA} + T_L + T_{DRB}\right) - i2\pi\nu_B\left(T_{CRB} + T_{DRB} + \tau_{BA}\right)\right] \\ &\times \sum_k E_{A,k}E_{B,k}\exp\left[i2\pi k\Delta f_r t + i2\pi k f_A\left(T_{CRA} + T_L + T_{DRB}\right) - i2\pi k f_B\left(T_{CRB} + T_{DRB} + \tau_{BA}\right)\right]\} \end{aligned} \tag{3}$$

The time information is extracted with high precision from the interference phase. By using the Fast Fourier Transform (FFT), the phase information Φ of the kth comb tooth can be calculated as:

$$\begin{aligned} \Phi_A\left(k, T_L\right) &= 2\pi\nu_A\left(T_{CRA} + T_{DRA}\right) - 2\pi\nu_B\left(T_{CRB} + T_L + T_{DRA} + \tau_{BA}\right) \\ &\quad + 2\pi k f_A\left(T_{CRA} + T_{DRA}\right) - 2\pi k f_B\left(T_{CRB} + T_L + T_{DRA} + \tau_{BA}\right) \\ \Phi_B\left(k, T_L\right) &= 2\pi\nu_A\left(T_{CRA} + T_L + T_{DRB}\right) - 2\pi\nu_B\left(T_{CRB} + T_{DRB} + \tau_{BA}\right) \\ &\quad + 2\pi k f_A\left(T_{CRA} + T_L + T_{DRB}\right) - 2\pi k f_B\left(T_{CRB} + T_{DRB} + \tau_{BA}\right) \end{aligned} \tag{4}$$

By subtracting $\Phi_A\left(k, T_L\right)$ from $\Phi_B\left(k, T_L\right)$, we get:

$$\begin{aligned} \Delta\Phi\left(k, T_L\right) &= 2\pi\left(\nu_A - \nu_B\right)\left(T_{DRB} - T_{DRA}\right) + 2\pi\left(\nu_A + \nu_B\right)T_L \\ &\quad + 2\pi k\left(f_A - f_B\right)\left(T_{DRB} - T_{DRA}\right) + 2\pi k\left(f_A + f_B\right)T_L \end{aligned} \tag{5}$$

The value of T_L can be calculated in the formula by using the phase change as the comb tooth serial number k increases. However, the presence of the term $2\pi k\left(f_A - f_B\right)\left(T_{DRB} - T_{DRA}\right)$ in the formula introduces an error due to the difference in path lengths between the reference plane and the detector at terminals A and B. In our device, the magnitude of $(T_{DRB} - T_{DRA})$ is approximately 1 ns, corresponding to a length difference of about tens of centimeters in the optical fibers within the two setups. According to formula (5), the direct impact of these two errors on the flight time T_L is further multiplied by a coefficient factor $M = (f_A - f_B)/(f_A + f_B) \approx 10^{-5}$. Therefore, their ultimate influence is that $M\left(T_{DRB} - T_{DRA}\right)$ is about 10 fs.

To cancel this error, we interchanged the values of the repetition rates f_A and f_B and merged both sets of data into a unified dataset. Because the peak-to-peak fluctuation $\Delta(T_{DRB} - T_{DRA})$ caused by environmental changes is around 1 ps, $M\Delta(T_{DRB} - T_{DRA})$ is about 0.01 fs. Data unification does not introduce a significant error. The interchange of the repetition frequency values does not affect the values of ν_A and ν_B. The phase is calculated as follows:

$$\Delta\Phi\left(k, T_L\right) = 4\pi(\nu_A + \nu_B)T_L + 4\pi k\left(f_A + f_B\right)T_L \tag{6}$$

To illustrate that the relative drift of the reference frequencies at sites A and B can be ignored after processing, the formula in this case is presented as follows. In each terminal, OFCs are phase-locked to the ultra-stable laser, and the repetition rates of the OFCs are uniquely determined by the frequency of the ultra-stable laser. In our experiment, the ultra-stable laser at site A is used as the frequency standard and treated as a fixed value. Considering the relative drift of the ultra-stable laser at terminal B, the repetition rate of the OFC at site B exhibits an offset. Thus, the repetition rate at site B is :

$$f_B^{'}(t) = f_B - \Delta(t) \tag{7}$$

Where f_B is the repetition rate when there is no relative drift of the ultra-stable laser at terminal B, and $\Delta(t)$ is the offset of repetition rate. Therefore, formula (4) is modified to:

$$\begin{aligned} \Phi_A\left(k, T_L, t\right) &= 2\pi\nu_A\left(T_{CRA} + T_{DRA}\right) - 2\pi\nu_B^{'}\left(T_{CRB} + T_L + T_{DRA} + \tau_{BA}\right) \\ &\quad + 2\pi k f_A\left(T_{CRA} + T_{DRA}\right) - 2\pi k f_B^{'}\left(T_{CRB} + T_L + T_{DRA} + \tau_{BA}\right) \\ \Phi_B\left(k, T_L, t\right) &= 2\pi\nu_A\left(T_{CRA} + T_L + T_{DRB}\right) - 2\pi\nu_B^{'}\left(T_{CRB} + T_{DRB} + \tau_{BA}\right) \\ &\quad + 2\pi k f_A\left(T_{CRA} + T_L + T_{DRB}\right) - 2\pi k f_B^{'}\left(T_{CRB} + T_{DRB} + \tau_{BA}\right) \end{aligned} \tag{8}$$

Where $\nu_B^{'}$ is the value of ν_B when there is a relative drift. The clock difference τ_{BA} arises from the additional phase of the OFC at site B, which is induced by the time-integration of the offset in repetition rate $\Delta(t)$. Therefore, the clock difference can be interpreted as $\tau_{BA} = 2\pi\int_0^t \Delta(\tau)d\tau/2\pi f_B^{'}$, and the phase information Φ is:

$$\begin{aligned}\Phi_A(k,T_L,t) =&2\pi\nu_A(T_{CRA}+T_{DRA})-2\pi\nu_B^{'}(T_{CRB}+T_L+T_{DRA}+\tau_{BA})\\&+2\pi kf_A(T_{CRA}+T_{DRA})-2\pi kf_B^{'}(T_{CRB}+T_{DRA})-2\pi kf_BT_L-2\pi k\int_0^t\Delta(\tau)d\tau+2\pi kT_L\Delta(t)\\\Phi_B(k,T_L,t) =&2\pi\nu_A(T_{CRA}+T_L+T_{DRB})-2\pi\nu_B^{'}(T_{CRB}+T_{DRB}+\tau_{BA})\\&+2\pi kf_A(T_{CRA}+T_L+T_{DRB})-2\pi kf_B^{'}(T_{CRB}+T_{DRB})-2\pi k\int_0^t\Delta(\tau)d\tau\end{aligned}\tag{9}$$

Obviously, $2\pi k\int_0^t\Delta(\tau)d\tau-2\pi kT_L\Delta(t)=2\pi k\int_0^{t-T_L}\Delta(\tau)d\tau$, and the phase information Φ can be finally calculated as:

$$\begin{aligned}\Phi_A(k,T_L,t) =&2\pi\nu_A(T_{CRA}+T_{DRA})-2\pi\nu_B^{'}(T_{CRB}+T_L+T_{DRA}+\tau_{BA})\\&+2\pi kf_A(T_{CRA}+T_{DRA})-2\pi kf_B^{'}(T_{CRB}+T_{DRA})-2\pi kf_BT_L-2\pi k\int_0^{t-T_L}\Delta(\tau)d\tau\\\Phi_B(k,T_L,t) =&2\pi\nu_A(T_{CRA}+T_L+T_{DRB})-2\pi\nu_B^{'}(T_{CRB}+T_{DRB}+\tau_{BA})\\&+2\pi kf_A(T_{CRA}+T_L+T_{DRB})-2\pi kf_B^{'}(T_{CRB}+T_{DRB})-2\pi k\int_0^t\Delta(\tau)d\tau\end{aligned}\tag{10}$$

It can be observed that the offset in the repetition rate of the OFC at site B has a certain influence on the results. However, by matching the data at different times, the data $\Phi_A(k,T_L,t+T_L)$, $\Phi_B(k,T_L,t)$ can be aligned to obtain results consistent with formula (5).

Owing to the high stability of the ultra-stable laser and calibration before experiment, the relative drift of ultra-stable lasers remains below 1 kHz during the experiment. Therefore, the offset of f_B is below 1E-3 Hz. For parameters such as T_{CRB}, T_{DRB}, T_{CRA}, and T_{DRA}, the direct impact from the offset in f_B is scaled by a coefficient factor $\Delta(t)/(f_A+f_B)$, which is on the order of 5E-12. Considering the magnitudes of T_{CRB}, T_{DRB}, T_{CRA} and T_{DRA} are approximately 10 ns, the resulting error can be ignored. To sum up, the clock difference between the two ultra-stable lasers has a negligible impact on the ranging accuracy.

2 Effects of atmospheric dispersion

The previous discussion on principles assumes a constant refractive index of air. However, in the actual atmospheric conditions, it is necessary to consider the multi-order relationship between the refractive index of air and optical frequency due to atmospheric dispersion. In fact, the relationship between $\Delta\Phi(k,T_L)$ and the serial number k of comb teeth is non-linear. The refractive index of air corresponding to k can be expressed as:

$$\begin{aligned}n(k)&=n_0+q(\upsilon-\upsilon_0)+p(\upsilon-\upsilon_0)^2+\mathrm{O}(\upsilon^3)\\&=n_0+qkf_r+p(kf_r)^2+\mathrm{O}(\upsilon^3)\end{aligned}\tag{11}$$

where n_0 represents the refractive index of air at the frequency ν_0. ν_0 is set as the frequency of the ultra-stable laser at sites A and B, as defined in our system. Because the optical frequency combs are phase-locked to ultra-stable lasers at beat frequencies $F_{beat}=\pm 35MHz$. Thus, in simple terms: $\nu_A=\nu_0-35MHz$, $\nu_B=\nu_0+35MHz$, $\nu_A+\nu_B=2\nu_0$, $n(\nu_A)+n(\nu_B)\approx 2n_0$. This approximation of the refractive index only introduces a small offset of about 1E-19, which can be safely ignored. q and p represent the first and second order coefficients of refractive index with respect to optical frequency. Based on Eqn. (6) and Eqn. (11), the phase $\Delta\Phi(k,L)$ can be calculated as follows:

$$\begin{aligned}\Delta\Phi(k,L)=&\frac{4\pi L}{c}[2n_0\nu_0+(n_0f_A+n_0f_B+q\nu_0f_A+q\nu_0f_B)k\\&+(q+p\nu_0)\left(f_A{}^2+f_B{}^2\right)k^2+p\left(f_A{}^3+f_B{}^3\right)k^3]\end{aligned}\tag{12}$$

The resulting phase in linear optical sampling can only be achieved within the interval $(0,2\pi)$. In fact, the range of distance L for each order coefficient of k is also limited by the phase range. We use d_0, d_1 and d_2 instead of L to represent the residues of different ambiguity ranges corresponding to the different order coefficients. Here, the distance interval for the zero-order coefficient is $(0,c/(4n_0\nu_0))$, which corresponds to an optical wavelength-level span. According to the air refractive index model [2], q is approximately 5.6×10^{-21} and p is about 1.5×10^{-35} at room temperature. Therefore, the interval of L for the first-order coefficient

becomes $(0, c/[2n_0(f_A + f_B)])$, corresponding to a distance range at (0, 0.3 m), while the range of the second-order coefficient is $(0, c/[2(q + p\nu_0)(f_A^2 + f_B^2)])$, corresponding to a distance range at (0, 1.4×10^{11} m). The coefficients of the third-order terms of k can be disregarded at approximately 100 km due to their negligible magnitude compared to the first and second order terms. Consequently, Eqn. (12) can be modified as follows:

$$\Delta\Phi\left(k, L\right) = \frac{4\pi}{c}\left[2n_0\nu_0 d_0 + \left(n_0 f_A + n_0 f_B + q\nu_0 f_A + q\nu_0 f_B\right) d_1 k + \left(q + p\nu_0\right)\left({f_A}^2 + {f_B}^2\right) d_2 k^2\right] \tag{13}$$

It is worth noting that the quadratic term of Eqn. (13) can be ignored when the measured distance falls within the range of millimeters to meters. However, as the absolute distance L extends from the laboratory path to the outdoor kilometer-level path, the ratio of the second-order coefficient to the first-order coefficient increases continuously. Therefore, a quadratic function needs to be used for fitting the relationship between $\Delta\Phi\left(k, T_L\right)$ and k.

Through the analysis of air dispersion, we can also obtain a rough distance value of the path to be measured. As shown in Fig. 1, fitting the phase curve with a quadratic function yields a distance d_2 of 113 km $\pm$ 2 km, as given by Eqn. (13).

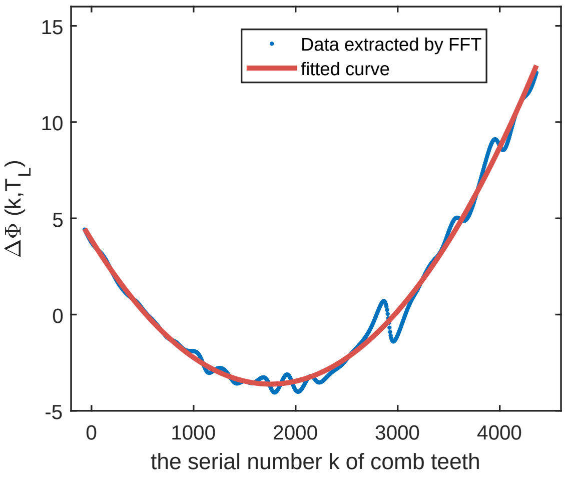

Figure 1: **The relationship between the serial number k of the comb teeth and the phase in combination with air dispersion analysis.**

3 Power loss of experimental path

The power loss of the entire transmission path, from the output of the EDFA at one terminal to the PD at the other terminal, can be calculated by using:

$$\eta = \eta_{fiber}\eta_{tele}T_{atm}\eta_{sm}\left(\frac{D_r}{L\theta}\right)^2 \tag{14}$$

where η_{fiber} represents the loss of local fiber paths at both terminals, which is approximately 4 dB. The loss of two telescopes, denoted as η_{tele}, is around 7.8 dB. Atmospheric transmittance T_{atm} [5], accounting for air absorption and scattering of the propagating beam along the path to be measured, is approximately 12.5 dB. η_{sm} represents the single-mode fiber coupling efficiency for free-space optical path through atmospheric turbulence [1, 4], which amounts to 27.9 dB in our systems. The geometric attenuation, expressed as $\left(D_r/L\theta\right)^2$, is 21.8 dB. The typical attenuation value for the entire path is 74 dB, but it may vary between a lower limit of 66 dB or an upper limit of 83 dB depending on weather conditions.

4 Power loss in inter-satellite ranging between GEO and LEO satellites

Here we calculate the geometrical loss between the GEO and LEO satellites using the bistatic method and the monostatic method, respectively. In the bistatic method, the geometrical loss can be represented as follows:

$$\eta_b = \left(\frac{D_t}{L\theta}\right)^2 \tag{15}$$

where D_t repersents the aperture of the telescope, L is the inter-satellite distance between GEO and LEO satellites, and θ represents the effective transmitter full-angle divergence. The values of the parameters could be estimated as $D_t = 400$ mm, $L = 36000$ km, $\theta = 5$ μrad, and the loss is calculated to be 53 dB. In monostatic method, the geometrical loss can be represented as follows:

$$\eta_m = \left(\frac{D_t}{L\theta}\right)^2 \left(\frac{D_m}{L\theta}\right)^2 \tag{16}$$

The aperture of the reflector D_m can be estimated to be the same as that of the telescope D_t, and the loss is calculated to be 106 dB.

5 Details of the experiment

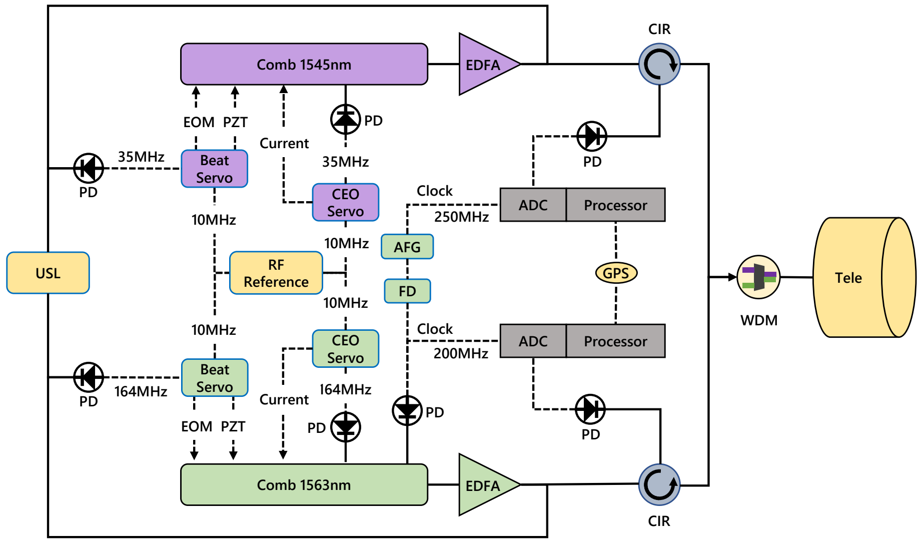

Figure 2: **Detailed experimental setup of a single terminal.** The solid lines represent the optical signals, while the dashed lines represent the electronic signals. Some abbreviations are: USL, Ultra-Stable Laser; EDFA, Erbium-Doped Fiber Amplifier; WDM, Wavelength-Division Multiplexer; GPS, Global Positioning System; ADC, Analog-to-Digital Converter; PD, Photodetector; CIR, Circulator; Tele, Telescope; FD, Frequency Divider; AFG, Arbitrary Function Generator; RF Reference, Radio-Frequency Reference.

Fig. 2 shows the experimental setup of one terminal. The setups for both terminals are nearly identical. Each terminal comprises two phase-locked OFCs with a spectral span of about 10 nm and a 1550.12 nm ultra-stable laser (USL). The upper part of the figure illustrates the 1545 nm OFC, while the lower part represents the 1563 nm OFC. The repetition frequency of the 1545 nm OFC at each terminal is set to 249.999 973 MHz ($f_{r,1545}$) or 249.999 973 MHz + 2.585 kHz ($f_{r,1545} + \Delta f_{r,1545}$), while the repetition frequency of the 1563 nm OFC is set to 199.995 018 MHz ($f_{r,1563}$) or 199.995 018 MHz + 2.068 kHz ($f_{r,1563} + \Delta f_{r,1563}$). The carrier-envelope offset (CEO) frequency and the beat frequency of OFCs, referenced to the radio-frequency reference (a rubidium atomic clock with an accuracy of 5E-11), are set to cancel each other out. Both OFCs use high-power EDFAs to amplify their power to approximately 1 Watt. The amplified signal is divided into two parts: one synchronized with the USL and the other used for ranging purposes. Circulators are used to distinguish

between transmitted and received signals. Most local fiber paths are contained within the integrated module, which maintains temperature stability with a standard deviation of about 10 mK.

The interferograms are digitized by a 14-bit analog-to-digital converter and recorded using a field programmable gate array (FPGA). Timing data are extracted using LOS, which includes Fast Fourier Transform (FFT), phase decoupling, and extraction of interference waveforms. The distance is calculated based on the timing data from the two sites. Electronic devices at each site are synchronized with USL through the repetition rate of the 1563 nm OFC. The start time of data collection at the two sites is within 30 ns using the Global Positioning System (GPS).

Group number	Parameters	Values
Group 1	$f_{r,1545,A}$	249.999 973 MHz
	$fr_{1545,B}$	249.999 973 MHz+2585.3 Hz
Group 2	$f_{r,1545,A}$	249.999 973 MHz+2585.3 Hz
	$fr_{1545,B}$	249.999 973 MHz
Group 3	$f_{r,1545,A}$	249.999 973 MHz
	$fr_{1545,B}$	249.999 973 MHz-2585.3 Hz
Group 4	$f_{r,1545,A}$	249.999 973 MHz-2585.3 Hz
	$fr_{1545,B}$	249.999 973 MHz

Table 1: Frequency for 1545 nm OFCs in a single synthetic repetition rate measurement

The correctness of the N_1 and N_2 is guaranteed by ensuring that each data point reaches the accuracy of $(D_{r2} - D_{r1})/4\sqrt{2}$. The extended ambiguity range and the desired accuracy can be adjusted by varying Δf_r. Table 1 presents four frequency groups for 1545 nm OFCs in a single measurement of synthetic repetition rate. To obtain sufficient accuracy, the acquisition time for each frequency group is set at 4 minutes. The change between frequency groups takes about 3 minutes. First, the beat frequency phase-locked loop of the comb is turned off, and then we monitor the repetition frequency with a frequency counter and adjust it by varying the voltage of the piezoelectric transducer. When the target frequency is reached, the beat frequency phase-locked loop is re-engaged to complete the process. The above process is carried out simultaneously at both sites. The total operation time for each measurement is half an hour.

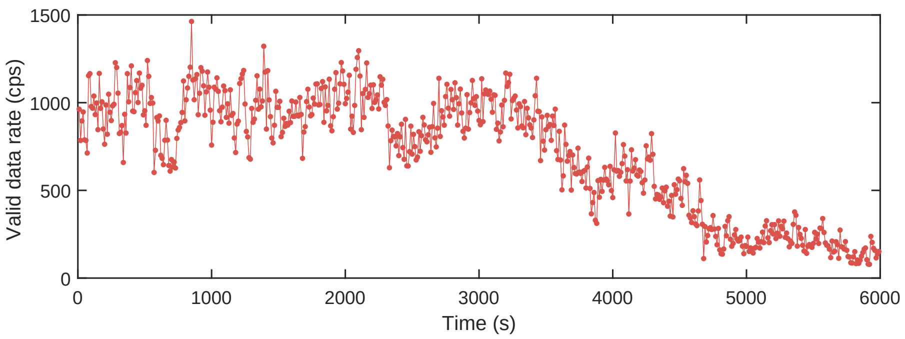

Figure 3: **The variation in the valid data rate during long-term ranging experiments.**

In our system, data are recorded only when the interference signal exceeds the preset intensity threshold. Therefore, data recording was mainly carried out during periods when atmospheric conditions were relatively favorable. We roughly assessed the weather conditions based on the data rate during the experiment. When the data rate drops below a certain level, data collection stops. Fig. 3 shows the variation in valid data rate during long-term ranging experiments, demonstrating our experimental strategy. The valid data rate is obtained by calculating the counts per second (cps) over a 10-second averaging period.

References

[1] Larry C Andrews and Ronald L Phillips. Laser beam propagation through random media. *Laser Beam Propagation Through Random Media: Second Edition*, 2005.

[2] Philip E Ciddor. Refractive index of air: new equations for the visible and near infrared. *Applied optics*, 35 (9):1566–1573, 1996.

[3] Ian Coddington, William C Swann, Ljerka Nenadovic, and Nathan R Newbury. Rapid and precise absolute distance measurements at long range. *Nature photonics*, 3(6):351–356, 2009.

[4] Yamaç Dikmelik and Frederic M Davidson. Fiber-coupling efficiency for free-space optical communication through atmospheric turbulence. *Applied Optics*, 44(23):4946–4952, 2005.

[5] John W Strohbehn. Laser beam propagation in the atmosphere. 1978.
